# Surgical treatment of spondylodiscitis in critically ill septic patients

**DOI:** 10.1007/s00701-023-05748-7

**Published:** 2023-08-17

**Authors:** Shadi Al-Afif, Oday Atallah, Dirk Scheinichen, Thomas Palmaers, Zafer Cinibulak, Jens D. Rollnik, Joachim K. Krauss

**Affiliations:** 1 Department of Neurosurgery, Hannover Medical School, Carl-Neuberg Str. 1, 30625 Hannover, Germany; 2 Department of Anaesthesiology and Intensive Care, Hannover Medical School, Hannover, Germany; 3 Institute for Neurorehabilitation Research (InFo), BDH-Clinic Hessisch Oldendorf, Affiliated Institute of Hannover Medical School, Hessisch Oldendorf, Germany

**Keywords:** Critical ill patient, Sepsis, Spinal surgery, Spondylodiscitis, Early surgery

## Abstract

**Purpose:**

Surgical procedures in critically ill patients with spondylodiscitis are challenging and there are several controversies. Here, we present our experience with offering surgical intervention early in critically ill septic patients with spondylodiscitis.

**Method:**

After we introduced a new treatment paradigm offering early but limited surgery, eight patients with spondylodiscitis complicated by severe sepsis and multiple organ failure underwent urgent surgical treatment over a 10-year period. Outcome was assessed according to the Barthel index at 12-month follow-up and at the last available follow-up (mean 89 months).

**Results:**

There were 7 men and 1 woman, with a mean age of 62 years. The preoperative ASA score was 5 in 2 patients, and 4 in 6 patients. Six of them presented with high-grade paresis, and in all of them, spondylodiscitis with intraspinal and/or paravertebral abscesses was evident in MR imaging studies. All patients underwent early surgery (within 24 h after admission). The median time in intensive care was 21 days. Out of the eight patients, seven survived. One year after surgery, five patients had a good outcome (Barthel index: 100 (1); 80 (3); and 70 (1)). At the last follow-up (mean 89 months), 4 patients had a good functional outcome (Barthel index between 60 and 80).

**Conclusion:**

Early surgical treatment in critically ill patients with spondylodiscitis and sepsis may result in rapid control of infection and can provide favorable long-term outcome. A general strategy of performing only limited surgery is a valid option in such patients who have a relatively high risk for surgery.

## Introduction

Spondylodiscitis is a heterogeneous but potentially life-threating condition which may cause a variety of neurological deficits [4, 29]. The estimated incidence in developed countries ranges between 4 and 24 per million [17, 48]. Its incidence has been increasing in the last four decades mainly secondary to the aging population in most countries worldwide [23, 42].

The clinical presentation of spondylodiscitis is highly variable ranging from severe back pain to acute and life-threatening sepsis or rapidly progressive neurological deficits. Spondylodiscitis can either be the primary focus of septicemia or result from septicemia originating from other infection sources, such as pneumonia, urinary tract infection, endocarditis, or soft tissue abscesses [19].

Due to its close anatomical proximity to the spinal cord and cauda, spondylodiscitis can lead to severe neurological deficits. This can occur through direct compression of the spinal neural tissues or by causing instability through destruction of the osseous and ligamentous spinal structures [19]. In such instances, urgent surgery has been advocated to avoid persistent disability. In addition, spondylodiscitis may also be complicated by severe sepsis and multiple organ failure including heart failure, renal failure, severe pneumonia, respiratory insufficiency, or septic shock [8]. Many patients who present with spondylodiscitis have a history of multiple co-morbidities which makes treatment challenging and which may have a negative impact on outcome.

Medical treatment with antibiotics is the first choice in most instances of spondylodiscitis [34]. Surgery is indicated in patients with neurological deficits and involves the decompression of neural structures, stabilization, and re-establishment of spinal alignment, surgical source control with debridement of necrotic tissues, and drainage of abscesses [4, 28].

Despite tremendous developments in surgical, anaesthesiological, and intensive care treatment in the last years, the therapy of spondylodiscitis in critically ill patients is still challenging and has been considered to be associated with high morbidity and mortality [15]. Only few studies have concentrated on surgical treatment in critically ill patients with spondylodiscitis [7, 12, 18, 20]. There is even less experience with surgical treatment in critically ill patients with spondylodiscitis and sepsis [18], since surgery often has been thought to be contraindicated, and the subject is still debated [32]. Nevertheless, more recent studies indicate that mortality may be even higher in such patients solely having conservative treatment [29]. Here, we report our experience in offering early surgical treatment in critically ill patients with spondylodiscitis and severe sepsis.

## Methods

Since 2010, a new paradigm was introduced in the Department of Neurosurgery at Hannover Medical School offering limited but early (within 24 h after admission) surgical treatment including decompression, sanitation of the spinal infection, and short segment stabilization when deemed necessary also to critically ill patients with spondylodiscitis developing sepsis regardless of age or the physical state as determined by the American Society of Anesthesiologists (ASA) score. Previously, such patients would have undergone medical treatment only and would have been offered surgery only in case they would have stabilized and survived sepsis.

Criteria to proceed with surgery was an unequivocal diagnosis of spondylodiscitis, rapid deterioration of the clinical condition (despite antibiotic treatment), and the presence of neurological deficits. Contraindications for surgery were accompanying active malignant diseases and severe coagulopathies.

Acute respiratory distress syndrome (ARDS) was quantified with the Horowitz index (H-Index) which evaluates pulmonary function in ventilated patients by dividing blood oxygen pressure (PaO2) by inhaled oxygen fraction (FiO2) to calculate the PaO2/FiO2 ratio [3]. According to the H-Index, ARDS is classified as mild (PaO2/FiO2 201–300 mmHg with PEEP ≥ 5 cmH2O), moderate (PaO2/FiO2 101–200 mmHg with PEEP ≥ 5 cmH2O), or severe (PaO2/FiO2 ≤ 100 mmHg with PEEP ≥ 5 cmH2O). When patients did not meet all criteria for ARDS, the term “respiratory insufficiency” was used.

For the present study, surgical and medical reports of all spinal surgeries performed over a 10-year period were screened to identify patients meeting the following criteria. Inclusion criteria were as follows: (1) patients with clinical manifestation of spondylodiscitis, epidural abscesses, and/or paravertebral abscesses; (2) evidence of spondylodiscitis and spinal infections on preoperative imaging studies; (3) manifestation of severe sepsis that necessitated treatment in the intensive care unit (ICU) with aggressive intravenous catecholamine and fluid administration; and (4) patients who underwent surgery early after admission with decompression of neural structures, stabilization in case of biomechanical instability, and/or source control of infection site. Exclusion criteria were as follows: (1) patients with spondylodiscitis without a need for ICU treatment, (2) patients with specific spinal infections such as tuberculosis, and (3) patients who had spondylodiscitis subsequent to prior spinal surgery.

The protocol of this retrospective study included review of all available clinical and imaging data including documentation on the course of rehabilitation and supplementing information on long-term outcome.

All patients underwent surgical treatment according to standard departmental surgical techniques as described in detail elsewhere [2, 9, 13, 44].

Clinical outcome was assessed by scheduled follow-up examinations at 3 months and at 12 months after surgery when possible. Further, information on recent follow-up was obtained via structured telephone interviews with the patients or their relatives. The performance in activities of daily living and the degree of independence of patients were evaluated using the Barthel index [30] with scores of 0–20 indicating “total” dependency, of 21–60 “severe” dependency, of 61–90 “moderate” dependency, and of 91–99 “slight” dependency.

## Results

Eight patients were identified who fulfilled the inclusion and exclusion criteria of the present study. There were 7 men and 1 woman. Their age at surgery ranged between 53 and 78 years (mean age, 62 years). Seven had two or more co-morbidities such as arterial hypertension, diabetes mellitus, coronary heart disease, valvular heart disease, cardiomyopathy, atrial fibrillation, pulmonary hypertension, and chronic obstructive pulmonary disease (COPD) (Table 1). While only two patients were primarily admitted to our tertiary care center, the majority of patients (6 patients) were treated initially at other hospitals and were referred for further surgical and medical/intensive care treatment.

**Table 1:** Demographics and clinical data of 8 critically ill patients with spondylodiscitis and sepsis

Pat. No	Sex	Age	Co-morbidities	Main Symptoms at presentation	Location/number of segments with spondylodiscitis	Locations of abscesses	CRP at admission (mg/L)	Preoperative bacterial isolation	Empirical antimicrobial therapy	Preoperative ASA-score	Time interval between admission and surgery in hours
1	M	64	HT, DM,CHD	Sepsis, ARDS (H-index 120 mmHg), paraparesis	Lumbosacral/2	Epidural, paraspinal	129	*Staphylococcus aureus* in blood culture	Piperacillin, Meronem	4	10
2	M	53	HT, DM, recent THP-surgery, adiposity	Sepsis, endocarditis, ARDS (H-index 104 mmHg), acute renal failure (anuria, hyperkalemia: 6 mmol/l), tetraparesis	Cervical/1	Epidural, paraspinal	177	*Streptococcus pneumonia* in blood culture and from knee puncture	Moxifloxacin, Meftriaxon, Clindamycin	4	17
3	M	59	HT, CD	Sepsis, respiratory insufficiency with pneumonia, acute renal failure (anuria, hyperkalemia: 5.5 mmol/l), paraparesis	Lumbosacral/1	Epidural, paraspinal	37	*Staphylococcus aureus* in blood culture	Piperacillin, Daptomycin	4	21
4	M	54	CHD, VHD, history of lymphoma with cervical radiation therapy	Sepsis, esophagus rupture and mediastinitis, ARDS (H-index 109 mmHg), tetraparesis	Thoracocervical/3	Epidural, paraspinal	137	-	Clindmycin, Piperacillin	5	6
5	M	57	Schizophrenia	Sepsis, respiratory insufficiency, high grade tetraparesis	Cervical/3	Epidural, paraspinal	235	-	Clindmycin, Piperacillin	4	5
6	M	71	DCM, AF, CHD, VHD	Sepsis, acute heart failure, ARDS (H-index 113 mmHg), acute renal failure (anuria, hyperkalemia: 5.8 mmol/l)	Thoracic/1	Paraspinal	45	*Staphylococcus aureus* in blood culture	Flocloxacillin, Rifampicin	4	19
7	F	78	Hep. C, AF, CKD, CRF	Sepsis, ARDS (H-index 124 mmHg), acute renal failure (anuria, hyperkalemia: 6.7 mmol/l), paraparesis	Lumbar/2	Epidural, paraspinal	34	*E*. *coli* in blood culture	Clindamycin, Cefepim	4	24
8	M	58	HT, PH, COPD, DM, AM, CHD, VHD, adiposity	Sepsis, endocarditis, respiratory insufficiency	Cervical/1	Epidural, paraspinal	128	*Staphylococcus aureus* in blood culture	Cefrtriaxon, Clindamycin	5	20

*ARDS* acute respiratory distress syndrome, *H-index* Horowitz index, *HT* arterial hypertension, *DM* diabetes mellitus, *CHD* coronary heart disease, *CRF* chronic renal failure, *VHD* valvular heart disease, *CM* cardiomyopathy, *AF* atrial fibrillation, *Hep. C* hepatitis C, *CKD* chronic heart disease, *PH* pulmonary hypertension, *COPD* chronic obstructive pulmonary disease, *THP* total hip prosthesis surgery

### Preoperative medical and neurological course

After admission to the ICU, all patients were treated with continuous intravenous catecholamines and high-load intravenous fluids to maintain sufficient mean perfusion blood pressure. The ASA score before surgery was 5 in 2 patients and 4 in 6 patients (Table 1).

**Fig. 1:**
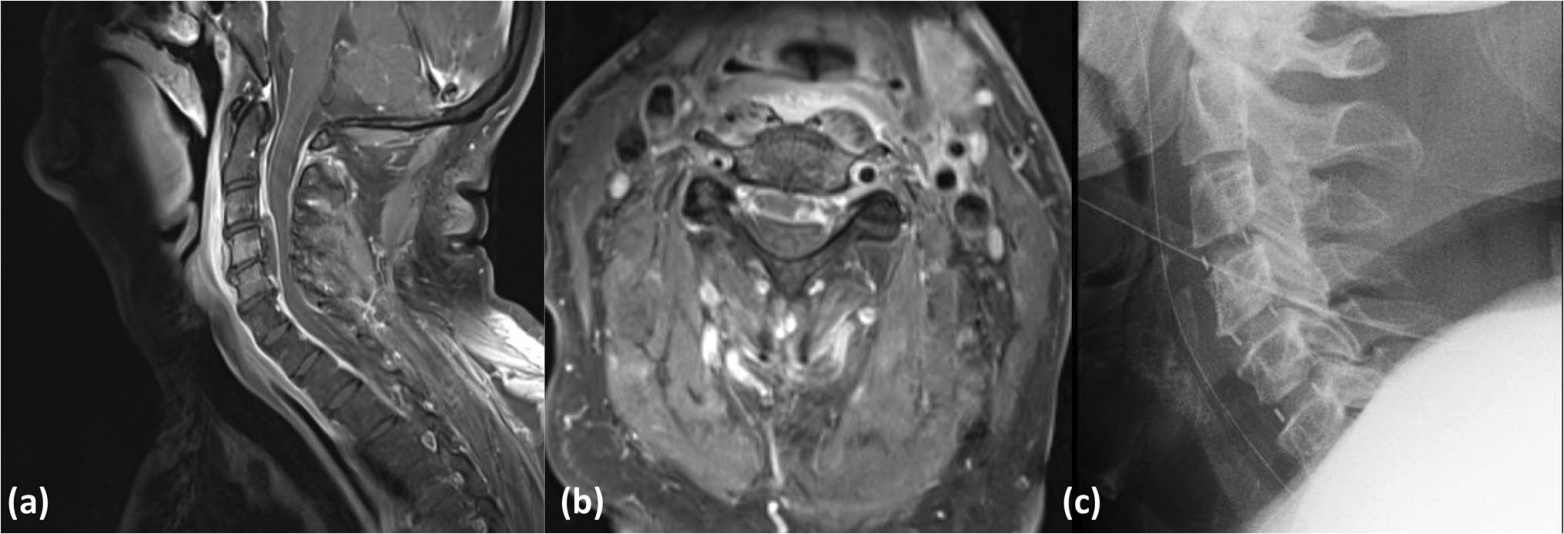
A 57-year-old man presented with progressive tetraparesis and severe sepsis. Sagittal and axial MRI (T1-weighted with gadolinium) shows cervical spondylodiscitis and an intraspinal longitudinal abscess with compression of the spinal cord (**a**, **b**). Emergency surgery was performed including ventral discectomy, decompression of the epidural abscess, and insertion of intervertebral cages at 3 cervical levels (**c**). During ICU treatment and antibiotic therapy, the tetraparesis resolved completely, and the patient was discharged to rehabilitation

All 8 patients had respiratory problems, 4 of them with the criteria of ARDS and 4/8 had multiple organ dysfunction (Table 1). Three were intubated for mechanical ventilation, and the other 5 received non-invasive ventilation therapy. In all patients, the c-reactive protein (CRP) was elevated (range 34–235 mg/L). After blood cultures had been taken, an immediate empirical antimicrobial treatment was initiated within the first hour of admission in all instances. The antibiotic regimen included a wide spectrum of antibiotics covering gram-positive and gram-negative bacteria (Table 1). Blood cultures were positive in 6 patients (75%) identifying the causal pathological organisms. In 5 instances, gram-positive bacteria were found (*Staphylococcus aureus* (*n* = 4), *Streptococcus pneumonia* (*n* = 1)), and in one patient, gram stains were negative (*E*. *coli*). According to the microbiological results, the antibiotic treatment was adjusted in 4 patients. In 3 patients, primary infection sites were identified (Table 1) including (1) infected knee implant (patient 2), (2) perforated esophagus and mediastinitis after endoscopic esophageal dilatation with a history of prior radiation treatment of cervical lymphoma (patient 4, Fig. 1), and (3) endocarditis (patient 8).

Preoperative neurological examinations revealed severe neurological deficits in 6 patients (Table 1) with paraparesis (3 patients) and tetraparesis (3 patients). Two patients had intact motor functions.

### Preoperative imaging studies

**Fig. 2:**
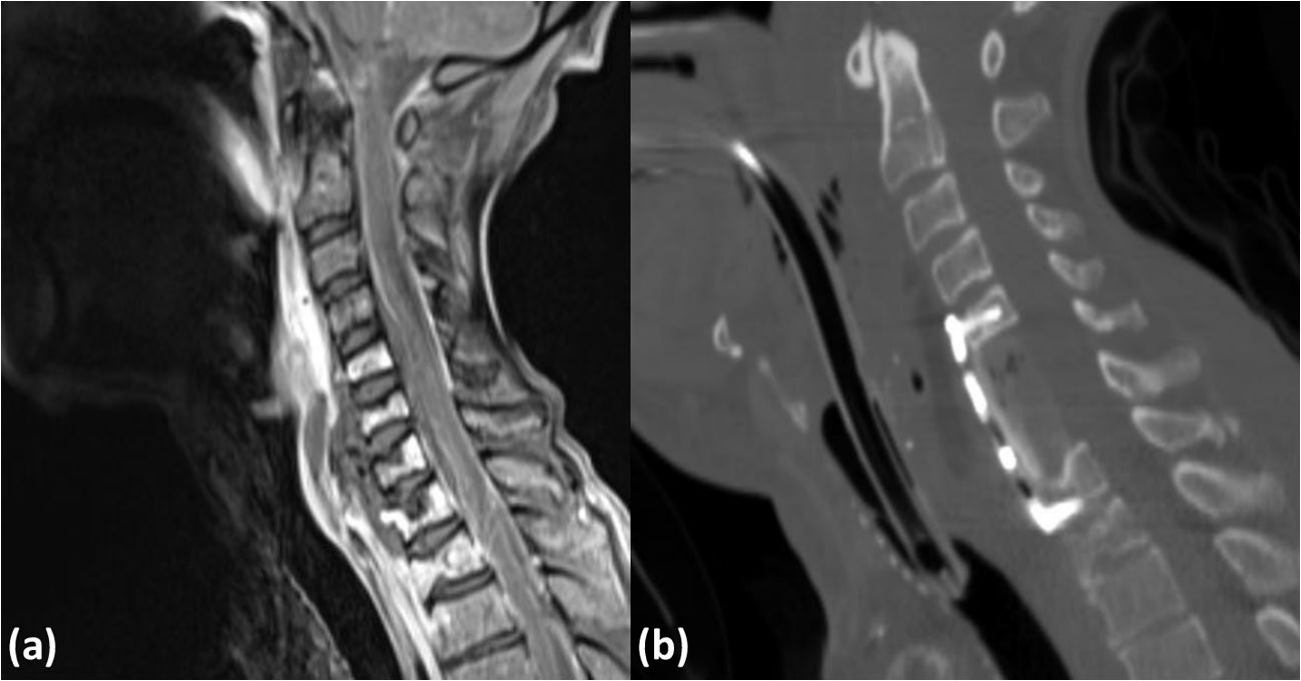
A 54-year-old man with tetraparesis and septic shock. Sagittal MRI (T1-weighted with gadolinium) demonstrates multilevel spondylodiscitis and massive prevertebral and epidural abscess in the lower cervical segments (**a**). Postoperative CT after emergency surgery including ventral corporectomy of C6 and C7, decompression of the epidural and prevertebral abscess, and fusion with autologous bone graft harvested from the ilium supplemented by plate osteosynthesis (**b**). The tetraparesis resolved partially prior to discharge for rehabilitation

Preoperative MRI with gadolinium confirmed spondylodiscitis in all instances. Spondylodiscitis was located in the cervical spine in 3 patients, in the thoracocervical spine in one, in the thoracic spine in another one, and in the lumbosacral spine in 3 patients (see Figs. 1 and 2). All patients had epidural infections with compression of the spinal cord, cauda, or spinal nerves in addition to paravertebral abscesses (Figs. 1 and 2).

### Surgical treatments

The surgical treatment strategy was adopted individually according to the medical and surgical circumstances in every patient. Preoperative correction of arterial hypotension and coagulation parameters was achieved in all patients. The time interval between admission and surgery for each patient is outlined in Table 1.

The surgical techniques which were applied included the following: (1) dorsal interlaminar decompression and drainage of epidural abscesses (2 patients); (2) cervical corpectomy, drainage of epidural abscesses, and fusion with autologous bone graft harvested from the iliac crest (2 patients) (Fig. 1); (3) anterior cervical discectomy, drainage of epidural abscesses, and fusion with PEEK-cage (2 patients) (Fig. 2); (4) dorsal laminectomy, drainage of epidural abscess, and dorsal fixation with transpedicular screws (1 patient); (5) transthoracic decompression of a prevertebral abscess (1 patient).

The mean duration of surgery was 117 min (range 87–257 min). In one patient, an accidental durotomy occurred (patient 1, Table 2), which was closed primarily with stitches, without a cerebrospinal fistula postoperatively. In patient 6, the surgery had to be stopped after drainage of the prevertebral abscess. The initially planned dorsal stabilization with pedicle screws was not possible due to severe intraoperative cardiopulmonary decompensation.

**Table 2:** Surgical treatment and postoperative courses of 8 critically ill patients with spondylodiscitis and sepsis

Pat. No	Surgery	Time of surgery in minutes	Intraoperative complications	Bacterial isolation obtained from surgery	Early (1–3 day) postoperative CRP	Adjustment of the antimicrobial treatment	Length of antimicrobial treatment in days: iv/oral (total period)	Postoperative complications in ICU	Length of ICU stay (days)	Length of hospital stay before rehab (days)	Neurological outcome at discharge	Neurological state (12 months after surgery)	Barthel-index (12 months after surgery)	Last follow up in months after surgery / Barthel-index at last follow-up
1	Dorsal lumbar, interlaminar decompression, and drainage of the epidural abscesses	205	Dural injury	*Staphylococcus aureus*	83	Clindamycin, Flucloxacillin	15/33 (48)	-	3	17	Improvement of paraparesis from grade 0/5 to 3/5	Improvement of paraparesis with residual footparesis (4/5)	70	174/50
2	Anterior cervical discectomy, drainage of the epidural abscesses, and fusion using PEEK-cage	292	-	-	168	Vancomycin, Meronem	87/30 (117)	Endocarditis, pneumonia, renal failure, septic encephalopathy	33	42	Improvement of tetraparesis from grade 1/5 to 4/5	No neurological deficits	100	48/ died 48 months after surgery due to heart infarction
3	Dorsal lumbar laminectomy, drainage of epidural abscesses, and dorsal fixation via transpedicular screws	150	-	-	61	Cephazolin	32/31 (63)	-	5	5	Improvement of paraparesis from grade 1/5 to 3/5	Mild gait ataxia	80	155/70
4	Cervical corpectomy, drainage of the epidural abscesses, and fusion with bone graft harvesting from iliac crest	230	-	*Streptococcus anginosus*, *Lactobacillus rhamnosus*, *Candida dubliniensis*, *Staphylococcus warner*	257	-	21/41 (62)	Reconstruction of esophagus after rupture	28	28	Improvement of tetraparesis from grade 0/5 to 3/5	Tatraparesis 3/5	15	15/ died 15 months after surgery due to pulmonary embolism
5	Anterior cervical discectomy, drainage of the epidural abscesses, and fusion using PEEK-cage	166	-	*Staphylococcus aureus*	64	Clindamycin, Flucloxacillin	49/44 (93)	-	9	17	Improvement of tetraparesis from grade 1/5 to 3/5	Tetraparesis 3/5	20	24/ died 24 months after surgery due to heart infarction
6	Transthoracic decompression of a prevertebral abscess	154	Severe cardiopulmonary deterioration, surgery must be abandoned	-	196	-	32/46 (78)	Acute pulmonal and cardial worsening	41	42	No neurological deficits, tracheostoma	No neurological deficits	80	98/60
7	Dorsal lumbar interlaminar decompression and drainage of the epidural abscesses	170	-	-	42	-	36/65 (101)	Reanimation, pneumonia. second revision surgery due to recurrance of epidural abscess	40	40	Improvement of paraparesis from grade 1/5 to 4/5	No neurological deficits	80	110/80
8	Cervical corpectomy, drainage of the epidural abscesses and fusion with bone graft harvesting from iliac crest	295	-	*Staphylococcus aureus*	66	Vancomycin, Meronem	24/33 (57)	Replacement of mitral valve, emergency revision surgery for hematothorax, pneumonia	11	107	Exitus letalis due to multiple organ failure	-	-	-

### Postoperative course and outcome

In the two patients with negative blood cultures, the microbiological cultures of samples obtained during surgery could identify the nature of the microorganisms. A second revision surgery was necessary for one patient due to recurrence of the epidural abscesses (patient 7, Table 2).

All patients were stabilized in the ICU postoperatively (median stay 21 days; range 3–41 days). The early postoperative course was complicated in 4 patients, and two of them underwent further secondary surgeries (reconstruction of a ruptured esophagus by ENT surgeons in patient 4 and replacement of a mitral valve and revision of thoracic hematoma in patient 8).

The duration of the antimicrobial therapy was adjusted according to the clinical course. Intravenous antibiotics were given at least for 2 weeks in all patients (median time intravenous treatment 37 days, range 15–87 days). The overall period of antibiotic treatment (intravenous and oral) was 11 weeks (range 48–117 days) (Table 2).

One patient passed away due to complications related to endocarditis on day 107 after spinal surgery. Seven patients survived and were referred to rehabilitation. At discharge, two had a tracheostomy and needed supportive ventilation. All 6 patients with motor deficits improved before referral to rehabilitation.

One year after surgery, five patients (70%) had achieved marked benefit (Barthel index: 100 (1 patient), 80 (3 patients), and 70 (1 patient). Two patients still needed intensive ambulatory care (Barthel index, 15 and 20, respectively) (Table 2). The mean long-term follow-up was 89 months (range 15–174 months). The distribution of the Barthel index with a favorable outcome ranged between 60 and 80 in four patients, and one patient had died due to myocardial infarction unrelated to septic spondylodiscitis (Table 2). The two patients who had a low Barthel index at 1-year follow-up, died later due to myocardial infarction and pulmonary embolism (15 and 24 months after surgery, respectively) (Table 2).

## Discussion

Our study shows that early surgical treatment in critically ill patients with spondylodiscitis and sepsis offers several advantages, and it may result in both rapid control of infection and favorable long-term outcome with improvement of neurological deficits in the majority of patients. While in the past, such patients often were not considered candidates for immediate surgery [38]; there has been a gradual change in opinion resulting in a call for earlier and more aggressive surgical treatment [29]. Such an approach is particularly justified, since the cause of death in the population under study has been primarily related to worsening of sepsis and subsequent progression of disease [29, 45]. In a previous study on spondylodiscitis, the number of deaths was significantly higher in patients with conservative treatment then in those who underwent early surgery. Remarkably, 59% of reported deaths were attributed to septic multiple organ failure [29]. We suggest that our study will further stimulate to consider a change of paradigm.

In a previous study on offering early surgery to patients with septic hematogenous lumbar spondylodiscitis in elderly patients by operative decompression and eradication of the intraspinal and intervertebral infective tissue with fusion via a posterior approach, there was a perioperative mortality of 17% and a morbidity of 50% [18]. With regard to their relatively high morbidity, the authors concluded that surgical treatment should not be the therapy of first choice in highly septic patients, but it may be considered when conservative management has failed [18]. While postoperative mortality was comparable in our study, morbidity was much lower when adopting a more flexible and limited surgical approach.

The complication rate in our study is comparable to another previous study on early surgery for spondylodiscitis [41]. In this study, 2 patients succumbed due to septic shock associated with endocarditis [41]. However, it was not specified how many patients overall presented with sepsis at the time of surgery. Remarkably, in our study in only one patient, surgery had to be aborted after decompression and before planned stabilization because of intraoperative anaesthesiological problems despite high preoperative ASA grades in all instances. In that case, the transthoracic approach certainly contributed as an additional risk factor.

Risk factors for the development of sepsis in patients with spondylodiscitis include advanced age, diabetes mellitus, renal failure, and an immunocompromised health state [1, 47]. In our study, the majority of the critically ill patients had a history of 2 or more co-morbidities. Using a large Japanese database, Akiyama et al. could identify an overall in-hospital mortality rate of 6% in patients with spondylodiscitis, but it was significantly higher in patients on hemodialysis (OR, 10.56), diabetes (2.37), liver cirrhosis (2.63), malignancies (2.68), and infective endocarditis (1).

The identification of the causal pathogenic organisms is one of the most important steps in the treatment of spondylodiscitis and sepsis. Blood cultures before initiation of antibiotic treatment have been advocated. The sensitivity of blood cultures ranges between 30 and 78% [33]. In our study, blood cultures yielded a diagnosis in 75% of cases. This result more likely is due to the higher sensitivity of blood cultures in patients with fever and septicemia [22]. In two patients with negative blood cultures, however, the causal pathological organisms could be identified only from samples from the surgical site. In our study, in 3 patients, the sepsis resulted from extraspinal infection sites, but the source of sepsis was not identified in the other patients. While the cause for infection of the spine remained unclear, the spondylodiscitis itself might have been the source for the worsening of the sepsis [28].

Comparable to the treatment of sepsis in other infectious disorders, the treatment principles of septic spondylodiscitis involve early administration of broad-spectrum antibiotics and maintenance of the cardiovascular circulation to obtain an adequate perfusion pressure [6, 37]. With that regard, all patients in our study received empirical broad-spectrum antibiotics immediately after obtaining blood cultures. Whereas the antibiotic treatment in patients with uncomplicated spondylodiscitis can be postponed until obtaining a sample from the infection site (surgically or CT-guided), in septic spondylodiscitis, the administration of antibiotics should be started within 1 h to reduce the mortality rate [37]. Since the course of sepsis may be complicated by multiple organ dysfunction, early referral to the ICU to allow mechanical ventilation and dialysis is pivotal [5, 27].

Another aspect to consider in the treatment of sepsis is the source control of the bacterial spread [31], which is mainly performed surgically. In general surgery, the timing of surgery for source control in unstable patients with severe sepsis due to different etiologies is controversial [46]. Although some authors postulate that patients initially should stabilize medically and then undergo surgery [32], the majority of studies and guidelines argue for early source control [37, 40, 43].

The extent of surgical treatment for infectious spondylodiscitis has been discussed controversially [18]. Different surgical approaches have been reported ranging from minimally invasive approaches without fusion and instrumentation such as percutaneous or endoscopic suction aspiration and drainage [21, 35] to more aggressive surgical procedures [11, 14, 24]. More extensive surgeries have included posterior instrumentation combined with anterior bone grafting or insertion of cages [11, 16, 25], anterior approaches with bone grafting [24] combined with anterior instrumentation [14], posterior approaches and instrumentation with discectomy and interbody bone grafts [26, 36], and eradication of the infected tissue and interbody fusion [10]. Additionally, both one-stage and two-stage dorsoventral operations were described in previous studies [16, 25, 39].

Limitations of our study are its retrospective nature, the relatively low number of patients, and lack of a (historical) control group, while its strengths include a defined surgical treatment paradigm (early surgery) and a long follow-up in all patients without attrition.

While our study was not designed to investigate the superiority of a certain surgical approach (decompression only versus additional stabilization), it shows that a general strategy of performing only limited and the least possibly invasive surgical treatment is a valid option in this fragile population of critically ill patients who have a relatively high risk for surgery.

## Data Availability

Data is available upon reasonable request.
